# Green Marine Collagen–Chitosan Composites with Biocompatible, Hemostatic, and Pro-Healing Performance

**DOI:** 10.1021/acsabm.5c02493

**Published:** 2026-02-26

**Authors:** Marcelo Assis, Diana Gabriela Nina Nina, Karolyne dos Santos Jorge Sousa, Mirian Bonifacio, Amanda de Souza, Mariana Carvalho Simões, Renata Granito, Flavia de Oliveira, Ana Claudia Muniz Rennó

**Affiliations:** Department of Biosciences, 28105 Federal University of São Paulo (UNIFESP), Santos SP 11015-020, Brazil

**Keywords:** marine biopolymers, chitosan–collagen composites, skin tissue engineering, biocompatibility, circular economy

## Abstract

Marine-derived biopolymers have emerged as sustainable alternatives to synthetic polymers for biomedical applications, offering both environmental benefits and intrinsic bioactivity. However, the development of multifunctional wound dressings that combine ecological sustainability with an enhanced biological performance remains a key challenge. In this study, type I collagen extracted from the skin of *Micropogonias furnieri* was incorporated into chitosan matrices of different molecular weights at 0%, 30%, and 50% to engineer composite films for skin repair. Structural and physicochemical characterization by Fourier-transform infrared spectroscopy (FTIR), polarized light microscopy, X-ray diffraction (XRD), and differential scanning calorimetry (DSC) revealed the preservation of collagen fibrillar organization and a progressive disruption of chitosan semicrystallinity, leading to more amorphous, flexible, and hydrogen-bonded networks as the collagen content increased. Biological assays demonstrated high cytocompatibility with L929 fibroblasts for all formulations with the 50% collagen composites significantly enhancing metabolic activity and cell proliferation. Redox analysis showed stable ROS levels and a moderate increase in the level of RNS, suggesting a controlled oxidative environment conducive to tissue regeneration. Functional performance was further confirmed by accelerated wound closure in scratch assays, reaching nearly 90% after 48 h for collagen-rich films. Hemocompatibility studies indicated a reduced coagulation time without hemolysis, while genotoxicity assessments confirmed the absence of DNA damage. Overall, the integration of marine collagen into chitosan matrices yields sustainable, biocompatible, hemostatic, and genetically safe biomaterials with enhanced regenerative performance, highlighting their strong potential for advanced wound-healing applications.

## Graphical abstract

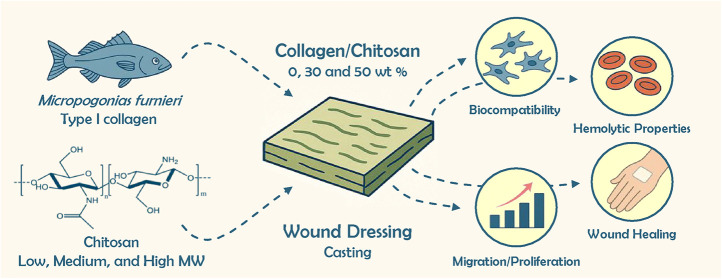

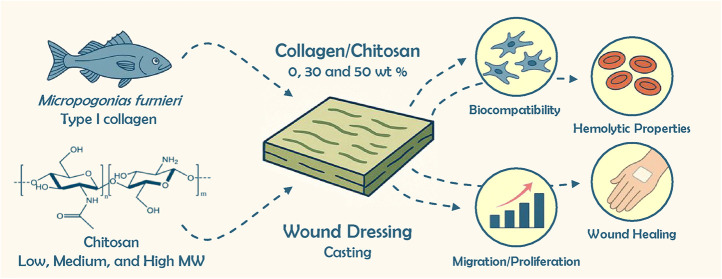

## 1 Introduction

In recent years, growing concern with environmental preservation has led to a shift in how we explore and utilize marine resources. Within the broader scope of the Ocean Decade, efforts have increasingly focused on identifying sustainable pathways for material development, particularly those that minimize environmental impact.^1^ The use of marine-derived substances, especially from renewable or low-waste sources, reflects a commitment to reducing dependence on synthetic or fossil-based inputs while promoting circular economy principles and contributing to the reduction of carbon footprint throughout the production cycle.^2^ This direction is strongly aligned with the principles of green chemistry, which emphasize the design of materials and processes that reduce or eliminate the use of hazardous substances and promote energy efficiency.^3^ By prioritizing cleaner production methods and biodegradable components, this approach also supports broader sustainability agendas, encouraging responsible innovation, lower emissions, and better integration between scientific progress and ecological stewardship.^4^ As interest in ocean-based solutions grows, so does the opportunity to develop safer, more sustainable technologies that contribute to both human health and marine conservation.

Building on this environmentally conscious perspective, the search for naturally derived biomaterials, especially those obtained from marine environments, has gained momentum in the field of regenerative medicine.^5^ Among the many clinical challenges, the effective treatment of skin wounds remains a complex issue, often limited by the lack of materials that combine biocompatibility, structural support, and sustainable sourcing.^6^ Traditional approaches still struggle to offer solutions that fully integrate with the healing process while minimizing the environmental impact. In this context, marine bioprospecting offers a valuable alternative, opening possibilities to identify novel matrices and structures capable of promoting tissue repair in a more balanced and responsible way.^7^ Exploring the vast diversity of marine ecosystems may yield materials that not only meet biomedical requirements but also align with broader goals of ecological responsibility and innovation in tissue engineering.

Biopolymers of natural origin have become key components in the design of membranes, films, and scaffolds for tissue engineering.^8^ Among them, chitosan stands out for its favorable biological profile and its origin from renewable marine sources, mainly the exoskeletons of crustaceans.^9^ It offers excellent biocompatibility, hemostatic activity, and intrinsic antimicrobial properties, which are especially relevant in wound-healing applications.^10^ One of the notable advantages of chitosan is the ability to tailor its properties through control of molecular weight.^11^ Low-molecular-weight chitosan tends to be more soluble and can diffuse more easily through biological tissues, which is useful in formulations for topical delivery or bioadhesive films. Medium-molecular-weight chitosan balances mechanical resistance with processability, while the high-molecular-weight form provides improved film-forming ability and structural integrity, which is particularly valuable for scaffold fabrication. These differences allow researchers to select or combine chitosan grades, depending on the mechanical demands and biological goals of the intended application. Zhang et al. observed that the mechanical stability of chitosan-based scaffolds improves with increasing molecular weight and also emphasized the importance of tailoring molecular weight according to the intended fabrication method.^12^

Fish collagen has gained increasing attention as a promising alternative to mammalian collagen, especially in contexts where sustainability, ethical sourcing, and reduced immunogenic risk are priorities.^13^ Extracted from byproducts of the fishing industry such as skins and scales, fish collagen contributes to the valorization of marine waste while offering functional benefits for tissue repair.^14^ It is particularly rich in type I collagen, the primary component of skin and connective tissue, and supports key biological processes including cell attachment, migration, and matrix remodeling.^15^ Its natural compatibility with dermal structures makes it suitable for use in various forms, from thin films and membranes to porous scaffolds that mimic extracellular environments. Another advantage lies in its biodegradability and ability to form hydrogels or blends with other polymers under mild processing conditions.^16^ Sousa et al. observed that collagen extracted from flounder fish (*Paralichthys* sp.) can stimulate the viability of HFF-1 and L929 fibroblast cells, with a more pronounced effect observed on the first day of exposure.^17^ In another study, Shalaby et al. evaluated collagen derived from tilapia and gray mullet scales in an *in vivo* rat model, reporting enhanced wound contraction as well as improved resolution and closure of skin injuries.^18^ These biological responses, combined with the environmentally sustainable origin of marine collagen, reinforce its potential as a promising candidate for the development of regenerative materials targeting both acute and chronic skin wounds.

Composites formed by the association of natural biopolymers have demonstrated great potential in tissue engineering, particularly when aiming to balance mechanical performance with biological functionality. The combination of chitosan and collagen has been explored due to the complementary nature of their properties.^19,20^ When used together, these polymers can produce matrices that are more stable, elastic, and cohesive than those prepared from either component alone. This synergy is especially valuable in skin regeneration, where materials must provide both structural support and biological compatibility. Chitosan contributes to the mechanical reinforcement and dimensional stability of the composite while also offering mild antimicrobial effects. Collagen, on the other hand, enhances the cellular response, promoting adhesion, proliferation, and tissue remodeling. The result is a material capable of mimicking the extracellular environment more effectively while maintaining the physical robustness needed during the healing process.

In this study, films composed of chitosan- and fish-derived collagen were developed with the aim of evaluating their potential for skin tissue engineering. The formulations were prepared by incorporating 0%, 30%, and 50% (*w*/*w*) fish collagen into chitosan matrices, and the effect of chitosan molecular weight (low, medium, and high) was also investigated. The collagen was extracted from the skin of croaker fish (*Micropogonias furnieri*), a marine species commonly found along the Atlantic coast. The collagen from other species, such as *Johniecop* spp., *Pseudosciaena* spp., *Nibea* spp., and *Larimichthys* spp., has already been extracted in various studies, showing promising results for biomedical applications.^21–27^ However, to date, there are no reports on the use of this particular species, which is commonly found and nonseasonal along the southern coast of Brazil. Its consistent availability and regional abundance make it an attractive and sustainable alternative source for biopolymer extraction. Film fabrication was carried out using the casting method, and the resulting materials were characterized by X-ray diffraction (XRD), differential scanning calorimetry (DSC), Fourier-transform infrared spectroscopy (FTIR), and mechanical analysis. Biological properties were assessed using murine fibroblasts (L929 cell line) focusing on metabolic activity, cell proliferation, adhesion, and migration assays. Additionally, intracellular levels of reactive oxygen species (ROS) and reactive nitrogen species (RNS) were quantified, and genotoxicity (using CHO-K1 cells) and hemocompatibility analyses were performed to provide a comprehensive evaluation of the biocompatibility and regenerative potential of the developed films.

## 2 Materials and Methods

### 2.1 Collagen Extraction

Fresh skins of croaker (*M. furnieri*), collected and processed under SISGEN authorization AA322F6, were first treated with 0.1 M sodium hydroxide (1:15 w/v, NaOH, Synth) at 4 °C under gentle agitation for 6 h, with the solution renewed every 2 h to remove noncollagenous proteins, cell debris, and other soluble residues. The material is rinsed with distilled water until reaching neutral pH. Defatting is then carried out using 10% (v/v) butanol (1:20 w/v, C_4_H_9_OH, Êxodo Cientifica) for 24 h at 4 °C, followed by thorough washing to eliminate residual lipids. The skins are subsequently depigmented with 3% (v/v) hydrogen peroxide (1:10 w/v, H_2_O_2_, Synth) for 15 min at low temperature, removing melanin and other pigments while contributing to asepsis, and again washed to neutral pH. Pretreated skins are frozen at −80 °C and lyophilized for 48 h to stabilize the collagen structure. Extraction is performed by incubating the dried material in 0.5 M acetic acid (1:50 w/v, CH_3_COOH, Synth) at 4 °C for 48 h under continuous agitation, followed by filtration to remove insoluble residues. Collagen is precipitated by adding sodium chloride (NaCl, Synth) to a final concentration of 2.5 M and stirring for 1 h, then separated by centrifugation (5000 rpm, 10 min, 4 °C). For final purification, the sample is dialyzed (12–14 kDa membrane, Sigma-Aldrich) in two stages: 48 h against 0.1 M acetic acid, followed by 24 h against 0.01 M acetic acid, both at 4 °C. This process removes residual salts, excess acid, and other low-molecular-weight impurities. The purified collagen solution is frozen at −80 °C, lyophilized for 72 h, and recovered as a high-purity dry collagen powder.

### 2.2 Composites

For the preparation of the composites, chitosan was dissolved at a concentration of 1 g/100 mL in 0.5 M acetic acid, using low- (Sigma-Aldrich, low molecular weight (<100 kDa), degree of deacetylation >75%, from shrimps), medium- (Sigma-Aldrich, medium molecular weight (100–300 kDa), degree of deacetylation >75%, from shrimps), and high-molecular-weight grades (Sigma-Aldrich, high molecular weight (>300 kDa), degree of deacetylation >75%, from shrimps). The same procedure was applied to the extracted collagen. The chitosan-to-collagen ratios were set at 0%, 30%, and 50% (*w*/*w*), as 50% was determined to be the minimum chitosan content required to produce a flexible, nonbrittle polymeric film. After obtaining the individual solutions, they were combined and homogenized using a mechanical stirrer at 1500 rpm for 5 min. The pH of the mixture was then adjusted to 6.0 using 0.1 M NaOH. Subsequently, 50 mL of the resulting solution was poured into a Petri dish and left to dry at 25 °C for 7 days. The dried films were carefully detached from the dish prior to further analysis. The prepared samples were identified according to the molecular weight of chitosan and the proportion of fish collagen incorporated. The codes CL, CM, and CH correspond to films based on low-, medium-, and high-molecular-weight chitosan. The suffix FC indicates the addition of fish collagen, while the numerical values represent the relative proportions of chitosan and collagen in the blend. Thus, 7CL3FC refers to a composition containing 70% chitosan and 30% fish collagen, and 5CL5FC corresponds to an equal ratio of both components. The same nomenclature applies to the medium- and high-molecular-weight chitosan formulations, resulting in the sets CM, 7CM3FC, 5CM5FC, CH, 7CH3FC, and 5CH5FC.

### 2.3 Characterization

FTIR spectra were recorded using a Jasco FT/IR-6200 spectrometer over the range of 600–4000 cm^–1^, with a total of 32 scans per sample. The crystalline structure of the materials was determined by XRD with a Shimadzu XDR-6100 diffractometer, employing Cu Kα radiation (λ = 1.54 Å). Data were collected in a 2θ range of 10–70°, with a scanning rate of 1°/min and a step size of 0.01°. DSC was performed on a Netzsch DSC 203 F3Maia instrument using 5–10 mg of sample. The thermal program consisted of heating from −50 to 400 °C at a constant rate of 10 °C/min. Tensile properties were evaluated in accordance with the ASTM D638 standard using a Shimadzu AGS-X universal testing machine (Japan) equipped with a 500 N load cell. Tests were performed at room temperature with a crosshead speed of 10 mm/min.

#### 2.3.1 Water Swelling

The films were dried at 60 °C for 24 h to remove any residual moisture. Each sample was then immersed in a Petri dish containing 50 mL of distilled water. At predetermined intervals, ranging from 1 to 7 days, the films were removed, gently blotted with absorbent paper to remove surface water, and weighed. The swelling percentage was calculated based on the difference between the wet and initial dry masses, normalized to the initial dry mass. All measurements were performed in quintuplicate to ensure reproducibility.

### 2.4 Biological Assays

#### 2.4.1 Cells

The L929 murine fibroblast and CHO-K1 (Chinese hamster ovary) cell lines were used in this study. The L929 cells were employed for cytocompatibility, oxidative stress, adhesion, and migration assays, while the CHO-K1 line was selected for the micronucleus test given its high sensitivity to genotoxic and clastogenic agents. All procedures were performed in compliance with the OECD Guidance Document on Good In Vitro Method Practices and ISO 10993-5:2009 for the biological evaluation of medical devices.^28^ L929 and CHO-K1 cells were maintained under the standard culture conditions. L929 cells were grown in Dulbecco’s Modified Eagle Medium (DMEM, VitroCell) supplemented with 10% heat-inactivated fetal bovine serum (FBS, VitroCell), while CHO-K1 cells were cultured in HAM F-12 medium (VitroCell) containing the same supplementation. All cultures were incubated at 37 °C in a humidified atmosphere with 5% CO_2_ until reaching approximately 80% confluence, with passaging performed as required to preserve cell viability and morphology.

Two experimental conditions were employed: direct contact and indirect contact. In the direct contact approach, sterile polymer films (10 mm) were placed at the bottom of the wells before seeding, ensuring a continuous interaction between the material surface and the cells. In the indirect contact approach, sample extracts were prepared by incubating the films in complete medium at a ratio of 0.1 g/mL for 24 h at 37 °C under humidified 5% CO_2_. The resulting extracts were filtered through a 0.22 μm membrane (Kasvi, Curitiba, Brazil) to remove residual particles and were applied to the cultures without further dilution. Control wells contained cells cultured without the test materials.

#### 2.4.2 Metabolic Activity

The viability of L929 murine fibroblast cells was evaluated by direct contact using resazurin (Sigma-Aldrich) as a metabolic activity indicator in compliance with ISO 10993-5:2009. Cells were cultured in DMEM (VitroCell) supplemented with 10% FBS and maintained at 37 °C in a humid atmosphere with 5% CO_2_. For the assay, cells were seeded at a density of 1 × 10^4^ cells per well in 500 μL of suspension in sterile 48-well plates and allowed to adhere for 24 h prior to exposure. Cell viability was assessed on days 1, 3, and 7. At each time point, a sterile resazurin working solution (prepared from a 0.1% w/v stock in PBS, filtered through a 0.22 μm membrane) was added directly to each well to reach a final concentration of 70 μM. Plates were incubated for 4 h at 37 °C in the dark to prevent photodegradation, and fluorescence was recorded at excitation/emission wavelengths of 560/590 ± 10 nm using a GloMax Discover (Promega) microplate reader. Raw fluorescence values were corrected for background and normalized to the mean of the negative control and set as 100% viability. According to ISO 10993-5:2009, viability values ≥ 70% were considered noncytotoxic. All experiments were performed in biological triplicates (*n* = 9), and the results were expressed as mean ± standard deviation.

#### 2.4.3 Proliferation

Cell proliferation was evaluated by direct contact quantifying the total DNA content at days 1, 3, and 7 after seeding for direct contact. For the assays, L929 cells were seeded in sterile 48-well plates at a density of 1 × 10^4^ cells per well in 500 μL of DMEM. At each time point, the culture medium was removed, and the wells were washed once with PBS. Cell lysis was performed by adding a buffer containing 10 mM Tris-HCl (pH 8.0, Synth), 10 mM EDTA (Neoquimica), 400 mM NaCl (Synth), and 1% (v/v) Triton X-100 (Sigma-Aldrich) in a sufficient volume to fully cover the scaffold. Plates were incubated at 37–55 °C for 2–4 h under gentle agitation. The resulting lysates were collected, and any remaining material in the well or on the scaffold was rinsed with small drops of PBS, which were pooled with the lysates. The samples were clarified by centrifugation at 5000 rpm for 10 min at 4 °C. DNA was precipitated with cold isopropyl alcohol (−20 °C), centrifuged at 5000 rpm for 15 min at 4 °C, washed with 70% ethanol, and air-dried for 15–20 min before being rehydrated in ultrapure water. DNA quantification was carried out using a NanoDrop spectrophotometer with the rehydration solution (nuclease free water, ThermoFisher) serving as the blank. Concentrations (ng/μL) were multiplied by the final sample volume to obtain the total DNA content per well and normalized by the control. All experiments were performed in biological triplicates (*n* = 9), and the results were expressed as mean ± standard deviation.

#### 2.4.4 ROS

L929 cells (1 × 10^4^ cells per well) were seeded into black 96-well culture plates and allowed to adhere under standard culture conditions. Experiments were conducted on days 1, 3, and 7 using the indirect contact method. At each time point, 100 μM H_2_DCFDA (Invitrogen) was added to each well, and the plates were incubated for 30 min at 37 °C in the dark. Fluorescence intensity was then recorded using a GloMax microplate reader (Promega) with excitation at 485 nm and emission at 530 nm. The results were expressed as relative fluorescence units and normalized against the untreated control cells. All experiments were performed in biological triplicates (*n* = 9), and the results were expressed as mean ± standard deviation.

#### 2.4.5 RNS

L929 fibroblast cells (1 × 10^4^ cells per well) were seeded into black 96-well culture plates and maintained under standard incubation conditions until adherence was achieved. The experiment was performed on days 1, 3, and 7 using the direct contact approach. At each time point, 50 μL of the culture supernatant was carefully collected and transferred to a separate plate. An equal volume (50 μL) of Griess reagent, comprising a 1:1 mixture of Solution A (1% sulfanilamide in 5% phosphoric acid) and Solution B (0.1% *N*-(1-naphthyl)­ethylenediamine dihydrochloride), was then added. The reaction was allowed to proceed for 15 min at room temperature, protected from light. Absorbance was read at 540 nm using a BioTek Instruments microplate spectrophotometer. Nitrite concentrations were quantified against a standard calibration curve prepared with known nitrite concentrations (nM), following the instructions of the modified Griess reagent kit (Sigma-Aldrich, G4410). All experiments were performed in biological triplicates (*n* = 9), and the results were expressed as mean ± standard deviation.

#### 2.4.6 Migration

The cell migration assay was performed by using an indirect contact approach. L929 cells were plated in 12-well culture plates at a density of 5 × 10^5^ cells per well and maintained for 24 h in DMEM supplemented with 1% FBS to stabilize basal cellular activity. A linear scratch was made across the central region of each well using a sterile 200 μL pipet tip guided by a sterile ruler. Detached cells and debris from the scratched region were removed by gently rinsing the wells with PBS. Subsequently, the cultures were exposed to conditioned media previously incubated with the films, allowing for the assessment of migration-promoting effects mediated by soluble factors released from the materials. Images were taken at 0, 24, and 48 h by using an inverted microscope equipped with a digital imaging system. The wound closure area representing cell migration was quantified using ImageJ software. All experiments were performed in triplicate (*n* = 3), and the results were expressed as mean ± standard deviation.

#### 2.4.7 Cell Adhesion

L929 fibroblast cells were seeded directly onto the surface of the scaffolds, which had been premoistened with complete culture medium (3 h), at a density of 1 × 10^6^ cells. The samples were incubated under standard culture conditions. Cell adhesion was evaluated 3 days after seeding by confocal laser scanning microscopy (SP8 AOBS Tandem Scanner, Leica Microsystems). Prior to imaging, scaffolds underwent a three-step wash with PBS to remove nonadherent cells. The adherent cells were fixed in a 4% paraformaldehyde (PFA, Synth) solution and subsequently stained with Phalloidin Alexa Fluor 488 (Invitrogen) to visualize actin filaments, while 4′,6-diamidino-2-phenylindole (DAPI, Invitrogen) was used to label DNA. The number of nuclei per square millimeter of the film was quantified using ImageJ software. All experiments were carried out in triplicate (*n* = 3), and the results are presented as the mean ± standard deviation.

#### 2.4.8 Genotoxicity

The micronucleus test was performed by indirect contact using the CHO-K1 cell line in accordance with previous work,^29^ with three independent experimental replicates to ensure reproducibility. Briefly, 0.5 × 10^6^ cells were seeded into six-well plates and allowed to adhere for 24 h under standard culture conditions. After the stabilization period, the cultures were treated with extracts obtained through indirect contact with the films to evaluate potential genotoxic effects mediated by soluble components. Following exposure, the medium was replaced with fresh DMEM containing Cytochalasin B (3 μg/mL, Sigma-Aldrich) and incubated for an additional 24 h to block cytokinesis. Cells were then washed twice with PBS, trypsinized, and centrifuged at 1500 rpm for 5 min. The resulting pellet was resuspended in a cold hypotonic solution (1% sodium citrate at 4 °C) for 4 min and fixed twice in methanol/acetic acid (3:1 v/v) under gentle centrifugation. The final suspension was dropped onto precleaned slides and air-dried before staining with a rapid panoptic kit (New Prov, Paraná, Brazil). The prepared slides were examined under a Nikon optical microscope at 630× magnification, and a minimum of 1000 binucleated cells per sample were scored for micronuclei. Negative control cultures received only the basal medium, and 40 μM mitomycin C was used as the positive control. All experiments were conducted in triplicate (*n* = 3), and the results are presented as the mean ± standard deviation.

#### 2.4.9 Hemostatic and Hemolytic Properties

This study was approved by the Research Ethics Committee of the Federal University of São Paulo (UNIFESP), registered on Plataforma Brasil (CAAE: 87066425.0.0000.5505). Written informed consent was obtained from all of the participants. Fresh citrated whole human blood (O^+^) was centrifuged at 200*g* for 15 min to separate plasma from erythrocytes. The collected plasma was then centrifuged again under the same conditions to concentrate platelets, followed by a third centrifugation at 1200*g* for 15 min. The upper fraction was collected as platelet-poor plasma (PPP), while the lower fraction was retained as platelet-rich plasma (PRP). To prepare platelet-deficient blood for the hemostatic assay, the erythrocyte pellet obtained from the first centrifugation was resuspended in an equal volume of PPP. For the hemostatic assay, 0.5 mL of platelet-deficient blood was added to each well of a 24-well plate containing the sample films, followed by 300 μL of 0.025 M CaCl_2_ solution to initiate coagulation. The samples were incubated at 37 °C, and the clotting time was monitored at short regular intervals by tilting the plate at 45° until stable clot formation was visually confirmed. After clot formation, 0.5 mL of distilled water was added to lyse the residual red blood cells. The color intensity of the supernatant, corresponding to the released hemoglobin, was measured as an inverse indicator of hemostatic efficiency (λ = 540 nm). Subsequently, the hemolytic activity of the samples was assessed by incubating 1 mL of citrated whole blood (1:5 in PBS) with the samples at 37 °C for 1 h followed by centrifugation at 150*g* for 5 min. The supernatant absorbance was measured at λ = 540 nm using a BioTek Instruments microplate spectrophotometer. Triton X-100 (0.1%) and PBS served as positive and negative controls, respectively. The hemolysis ratio (%) was determined according to eq 1

1 $$\mathrm{hemolysis}\;\left(\%\right)=\frac{\mathrm{Abs}\;\mathrm{sample}-\mathrm{Abs}\;\mathrm{negative}}{\mathrm{Abs}\;\mathrm{positive}-\mathrm{Abs}\;\mathrm{negative}}\times 100$$

All experiments were conducted in five replicates (*n* = 5), and the results are presented as the mean ± standard deviation.

#### 2.4.10 Statistical Analysis

Statistical analyses were carried out using GraphPad Prism 8.0 (GraphPad Software, San Diego, USA). Data were evaluated for normality prior to analysis, and differences among groups were assessed using one-way ANOVA followed by Tukey’s post hoc test for multiple comparisons. A value of *p* < 0.05 was considered statistically significant.

## 3 Results and Discussion

### 3.1 Structural Characterizations

Initially, the integrity of the collagen extracted from the skin of *M. furnieri* was evaluated to ensure its structural preservation prior to blending with chitosan, yielding 11.4 ± 2.7% based on the dry weight of the raw fish skin (Figure S1). The FTIR spectrum revealed the characteristic vibrational bands of collagen, particularly those corresponding to the amide I, II, and III regions, which are associated with the stretching and bending vibrations of C=O, N–H, and C–N bonds.^30^ These bands confirm the maintenance of the molecular framework of type I collagen after the acid extraction process. Confocal microscopy using Rhodamine B fluorescent labeling further evidenced the typical fibrillar architecture of collagen, revealing elongated and interconnected fibers.^31^ In addition, polarized light optical microscopy with Picrosirius staining showed yellow to reddish birefringence, confirming the predominance of type I collagen fibers.^32^ Together, these results demonstrate that the acid extraction procedure successfully yielded structurally intact type I collagen suitable for subsequent blending with chitosan of different molecular weights. The prepared blends aimed to assess, both structurally and biologically *in vitro*, the influence of chitosan molecular weight and collagen content on the properties of the membranes produced by casting. Collagen concentrations were fixed at 30% and 50% (*w*/*w*), as higher proportions tend to generate fragile films that easily fracture, making them unsuitable for handling and further testing.

#### 3.1.1 XRD

XRD analysis was first conducted to evaluate the crystalline organization of chitosan and to verify how collagen incorporation modifies its structural order (Figure 1A). The diffractograms of pure chitosan films exhibited three characteristic reflections centered at approximately 12°, 18°, and 24°, which correspond to the hydrated crystalline region, the semicrystalline packing of chitosan chains, and the amorphous scattering domain of the polysaccharide.^33^ The peak near 12° (2θ) is typically assigned to the hydrated crystal lattice formed by intermolecular hydrogen bonds between amino and hydroxyl groups, while the 18° reflection represents the regular arrangement of the polymer chains in the crystalline region. The broader signal around 24° is attributed to amorphous zones, where chain packing is disordered. These results are similar to that obtained by Qiao et al.^34^ Upon the addition of fish collagen, the diffraction peaks became less intense and lost definition, particularly the one at 18°, indicating a decrease in crystallinity and disruption of the ordered domains. This effect can be attributed to the insertion of collagen molecules between the chitosan chains, which interferes with the interchain hydrogen-bonding network and prevents the formation of well-organized crystalline lamellae. The loss of diffraction intensity thus reflects the amorphization of the matrix, suggesting a stronger molecular mixing between both polymers.

**Figure 1:**
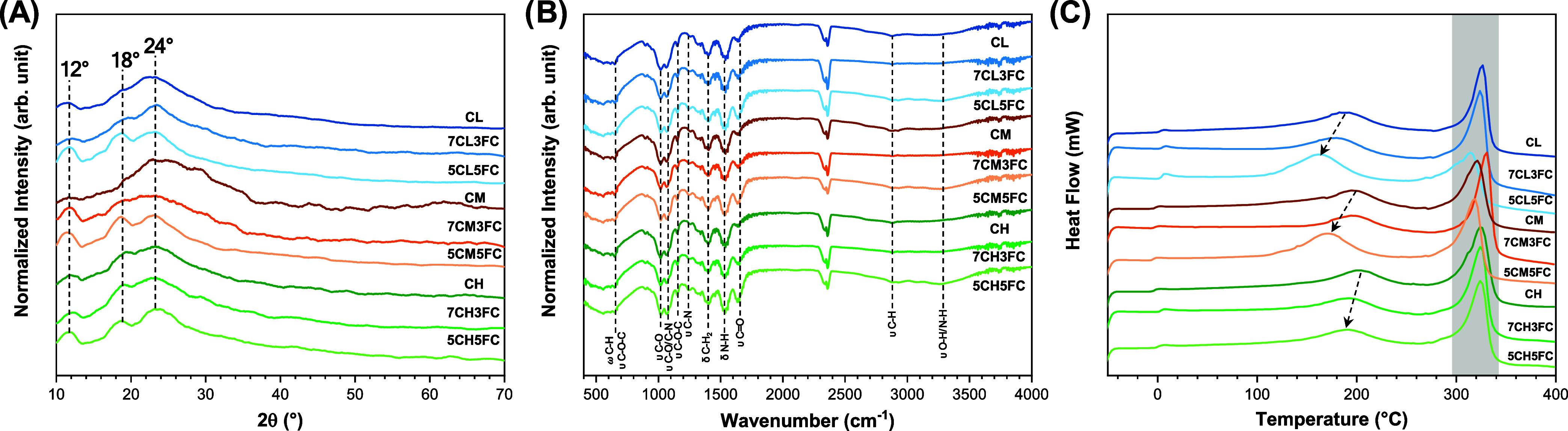
Characterization of chitosan- and collagen-based samples: (A) X-ray diffraction (XRD) patterns, (B) Fourier-transform infrared (FTIR) spectra, and (C) differential scanning calorimetry (DSC) thermograms.

#### 3.1.2 FTIR

FTIR spectroscopy was used to investigate possible chemical interactions between chitosan and collagen and to confirm the preservation of functional groups responsible for biological activity (Figure 1B). All samples exhibited characteristic absorption bands of chitosan/collagen at 648, 1021, 1064, 1155, 1241, 1403, 1537, 1642, 2879, and 3280 cm^–1^.^35–37^ These correspond to skeletal vibrations of the saccharide structure (C–O–C and C–O stretching), CH bending, and amide-related vibrations. The bands at 1642 cm^–1^ (amide I) and 1537 cm^–1^ (amide II) are associated with C=O stretching and N–H bending vibrations, reflecting the presence of acetylated groups and confirming the partial deacetylation typical of chitosan. The broad band between 3200 and 3400 cm^–1^ is attributed to overlapping O–H and N–H stretching vibrations, indicating extensive hydrogen bonding within the matrix. When collagen was added, no significant spectral shifts or new bands were observed, suggesting that no covalent bonding occurred between the polymers. Instead, the interaction is mainly physical, governed by hydrogen bonds and electrostatic attractions between the amino and carboxyl groups of both biopolymers.^38^ Therefore, the presence of interactions detected by FTIR indicates molecular affinity between the polymers but does not necessarily imply formation of a densely packed or mechanically reinforcing network. This is expected because the characteristic peaks of collagen, especially those of amide I, II, and III, overlap with the corresponding regions of chitosan, masking subtle shifts that might occur due to hydrogen bond formation.^39^ These results confirm that collagen is well-dispersed within the chitosan matrix, forming a blended system stabilized by noncovalent interactions with partial disruption of the original chitosan–chitosan intermolecular organization.

#### 3.1.3 DSC

DSC analysis was performed to assess how collagen incorporation affects the thermal transitions and molecular stability of the films (Figure 1C). Pure chitosan samples exhibited two main endothermic transitions. The first, located around 218 °C, corresponds to the breaking of intramolecular and intermolecular hydrogen bonds and to the evaporation of strongly bound water molecules within the polymeric network.^40^ This transition is commonly interpreted as relaxation of the crystalline domains and the onset of structural rearrangement. The second, broader transition between 320 and 340 °C is related to the thermal degradation of the polymer backbone, including deacetylation and depolymerization of the glucosamine units, as observed by Villegas-Peralta et al.^41^ Interestingly, the endothermic peak at ∼100 °C correspondent to the evaporation of water chitosan films is not observed, showing that the films are completely dried.^42^ After the addition of collagen, both transitions shifted to lower temperatures, indicating a decrease in the thermal stability and cohesive energy of the composite network. The reduction was more pronounced for films produced with low- and medium-molecular-weight chitosan, where the first transition appeared near 180 °C, evidencing higher chain mobility and weaker intermolecular bonding. This behavior suggests that collagen acts as a plasticizing agent, decreasing the rigidity of the chitosan chains and facilitating thermal relaxation.^43,44^ The high-molecular-weight samples retained higher transition temperatures, consistent with stronger chain entanglement and a more compact network. The downward shift of both transitions confirms that collagen disrupts the regular crystalline packing of chitosan, as previously observed in XRD, corroborating the formation of a less ordered and thermally softer system.

#### 3.1.4 Mechanical Analysis

Mechanical testing was then performed to evaluate the macroscopic effects of these molecular modifications. The tensile strength (TS) and elongation at break (EB) values obtained for all formulations are summarized in Table 1. In all molecular weights, a gradual reduction in TS was observed with an increasing collagen concentration. This indicates not the absence of intermolecular interactions but a redistribution of hydrogen bonding from ordered chitosan–chitosan domains to less organized chitosan–collagen contacts, which weakens the mechanical cohesion of the material. For example, in low-molecular-weight chitosan, TS decreased from 21.22 ± 1.97 MPa in the pure film to 19.58 ± 5.22 and 16.39 ± 4.69 MPa for 30% and 50% collagen, respectively. A similar behavior was recorded for the medium and high-molecular-weight formulations, although the decline was less pronounced in the latter due to the higher chain interconnectivity. This trend is aligned with the previous work of Hou et al. and Andonegi et al. that analyzes the mechanical properties of collagen–chitosan films.^44,45^ The EB exhibits a more complex trend. In general, collagen addition led to a slight decrease in EB, consistent with the reduction of cohesive interactions and the introduction of microheterogeneities that act as stress concentration sites. Thus, although FTIR confirms the existence of hydrogen bonding, these interactions become less spatially organized and less effective in load transfer, resulting in a lower mechanical resistance. The combined results demonstrate that the extent of these changes depends not only on the collagen content but also on the molecular weight of chitosan, which defines the degree of order and interchain cohesion in the matrix.

**Table 1:** Mechanical Tensile Properties of Chitosan- and Collagen-Based Samples, Including the Tensile Strength (TS) and Elongation at Break (EB)^a^

	low		medium		high	
collagen (%)	TS (MPa)	EB (%)	TS (MPa)	EB (%)	TS (MPa)	EB (%)
0	21.22 ± 1.97	4.65 ± 1.09	26.12 ± 2.98	4.24 ± 2.23	32.32 ± 3.24	3.28 ± 1.14
30	19.58 ± 5.22	4.07 ± 1.68	21.85 ± 5.10	4.19 ± 2.75	26.87 ± 6.94*	3.65 ± 2.52
50	16.39 ± 4.69*	3.83 ± 2.01	19.26 ± 4.46*	4.01 ± 2.54	21.60 ± 5.75**	3.08 ± 1.85

a The results are presented as mean ± standard deviation (*n* = 6). Statistical significance was determined by one-way ANOVA with post hoc test, where *p < 0.05 in relation to pure chitosan films.

#### 3.1.5 Water Uptake

The water uptake behavior revealed clear differences among the films as a function of the chitosan molecular weight and collagen content (Figure S2). Blends prepared with low-molecular-weight chitosan exhibited the highest swelling ratios, reaching values ∼460% (for 5CL5FC sample) and then showing a small decrease after the apparent equilibrium was reached. This behavior is related to the relaxation of the hydrated network and the release of loosely bound water. In addition, part of the soluble collagen and chitosan diffuses into the medium, reducing the measured wet mass and consequently the swelling percentage. This pronounced hydration capacity is attributed to the shorter polymer chains, which reduce intermolecular entanglement and facilitate water diffusion through the network.^46^ The incorporation of fish collagen further enhanced this effect by introducing additional hydrophilic groups, increasing chain mobility and hydrogen-bonding sites.^47^ In contrast, films produced with medium- and high-molecular-weight chitosan showed lower overall water uptake, as their denser polymeric networks restricted solvent penetration. The swelling kinetics of these systems reached equilibrium faster and declined more gently over time, indicating a more stable structure after hydration. These results confirm that the molecular architecture of chitosan plays a decisive role in defining the hydration and diffusion properties of the collagen–chitosan composites.

### 3.2 *In Vitro* Assays

#### 3.2.1 Metabolic Activity

After the successful extraction of type I collagen and the fabrication of homogeneous composite films were confirmed, *in vitro* biological assays were performed to evaluate their biocompatibility and cellular response. Murine fibroblast L929 cells were selected as the biological model because they are widely accepted in ISO 10993-5 cytocompatibility standards. These cells are highly sensitive to variations in surface chemistry and provide a reliable indicator of material-induced cytotoxicity, adhesion capacity, and proliferation potential. Metabolic activity was assessed by the alamar Blue assay, which reflects mitochondrial function and, therefore, the general cellular health and redox balance of the culture (Figure 2A–C).^48^ It is important to emphasize that metabolic activity does not necessarily equate to cell proliferation: while proliferation indicates an increase in cell number, metabolic activity reflects the efficiency of intracellular energy production and viability at a given time.^29^ Hence, both parameters were analyzed independently to better understand how the material influences cell function. On day 1 (Figure 2A), all groups demonstrated good cytocompatibility, with cell viability remaining above 90%. A small but statistically significant decrease was observed in the CH group (high-molecular-weight chitosan, ∼96%), suggesting that denser matrices may limit nutrient and oxygen diffusion during initial contact. By day 3 (Figure 2B), this trend persisted for CH (∼93%), whereas a clear stimulatory effect emerged for the low-molecular-weight chitosan formulations containing collagen (7CL3FC and 5CL5FC), which showed significantly increased metabolic activity (∼102–104%). On day 7 (Figure 2C), the differences became more pronounced: CH maintained slightly reduced activity (∼91%), while the 7CL3FC and 5CL5FC groups exhibited significantly enhanced metabolic activity (∼106–111%). The results indicate that incorporating fish collagen into low-molecular-weight chitosan films produces a softer and more hydrated structure. This characteristic facilitates nutrient and gas diffusion through the matrix, helping to maintain an environment that supports cellular metabolism. The addition of collagen also appears to improve the interaction between the cells and the material surface, promoting better adhesion and contributing to the overall maintenance of metabolic activity compared with pure chitosan films.

**Figure 2:**
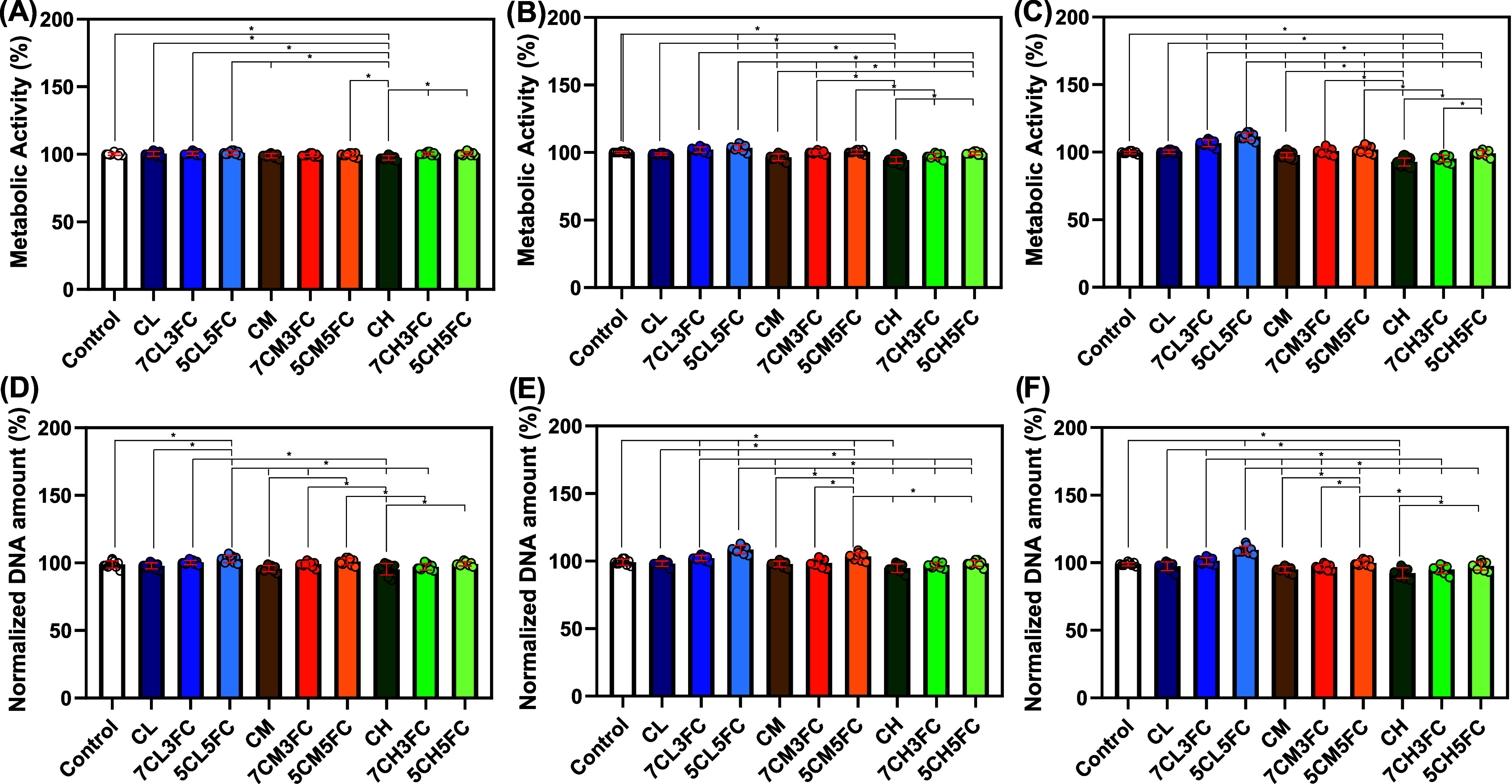
Cellular activity and proliferation of chitosan- and collagen-based samples. (A–C) Metabolic activity assessed by Alamar Blue assay at days 1, 3, and 7, respectively. (D–F) DNA quantification at days 1, 3, and 7, respectively. Data were normalized to control. The results are presented as mean ± standard deviation (*n* = 9). Statistical significance was determined by one-way ANOVA with post hoc test, where *p < 0.05.

#### 3.2.2 Proliferation

Cell proliferation was quantified by DNA content analysis, providing a direct measure of cell division. On day 1 (Figure 2D), only the 5CL5FC sample showed a statistically significant increase (∼102%) relative to the control, suggesting that a high collagen content accelerated early adhesion and spreading. By day 3 (Figure 2E), this trend persisted for 5CL5FC (∼107%) and extended slightly to 5CM5FC (∼103%), indicating that both low- and medium-molecular-weight chitosan can support fibroblast proliferation when combined with collagen. At day 7 (Figure 2E), these effects became more evident, with the 5CL5FC formulation maintaining the highest proliferation (∼108%), followed by a modest but consistent value for CH (∼90%). These results are in accordance with previous works that show collagen sources can improve cell proliferation.^49,50^ Ullah et al. show that chitosan/fish collagen (from tilapia scales) porous scaffolds can improve the fibroblast cell proliferation at longer times (4 and 7 days).^51^ In another work, Binlateh et al. observed that the 1:1 mixture of chitosan and collagen also improves cell proliferation.^52^ These findings reveal that metabolic activation precedes and promotes proliferative behavior, reflecting the ability of sample 5CL5FC to sustain cell energy metabolism and support mitotic activity over time.

#### 3.2.3 ROS and RNS

To further explore how the materials modulate intracellular signaling, ROS and RNS act as secondary messengers in pathways related to cell migration, differentiation, and immune modulation (Figure 3).^53^ Although excessive ROS can cause oxidative stress and damage macromolecules, moderate ROS levels are essential for initiating the inflammatory and proliferative phases of wound healing.^54^ The intracellular ROS analysis by DCFDA revealed comparable levels among most samples, with only minor yet statistically significant increases for CH and 7CH3FC at all experimental times (Figure 3A–C). These moderate elevations suggest a controlled oxidative response rather than cytotoxicity, consistent with active but nonstressed cell metabolism.^55^ RNS, primarily nitric oxide (NO) and related derivatives, play equally crucial but distinct biological roles. NO is known to regulate vascular tone, stimulate fibroblast migration, and promote angiogenesis, all key processes in tissue regeneration.^56^ The results demonstrated a clear concentration-dependent increase in the RNS for low-molecular-weight chitosan (CL) films containing collagen (Figure 3D–F). This effect intensified with higher collagen content, reaching peak values close to 4 nM on day 7 for the 5CL5FC formulation. Such enhancement in RNS production indicates a favorable redox environment that encourages collagen synthesis and cellular communication, without inducing oxidative stress.^57^ Lee et al. previously reported that low nitric oxide concentrations (approximately 1–30 nM) can stimulate angiogenesis, enhance cell survival, and promote proliferation by activating soluble guanylate cyclase (sGC) and consequently elevating intracellular cGMP levels.^58^ The interplay between ROS and RNS signaling is essential as balanced levels of these species orchestrate the transition from the inflammatory to the regenerative phase of healing.

**Figure 3:**
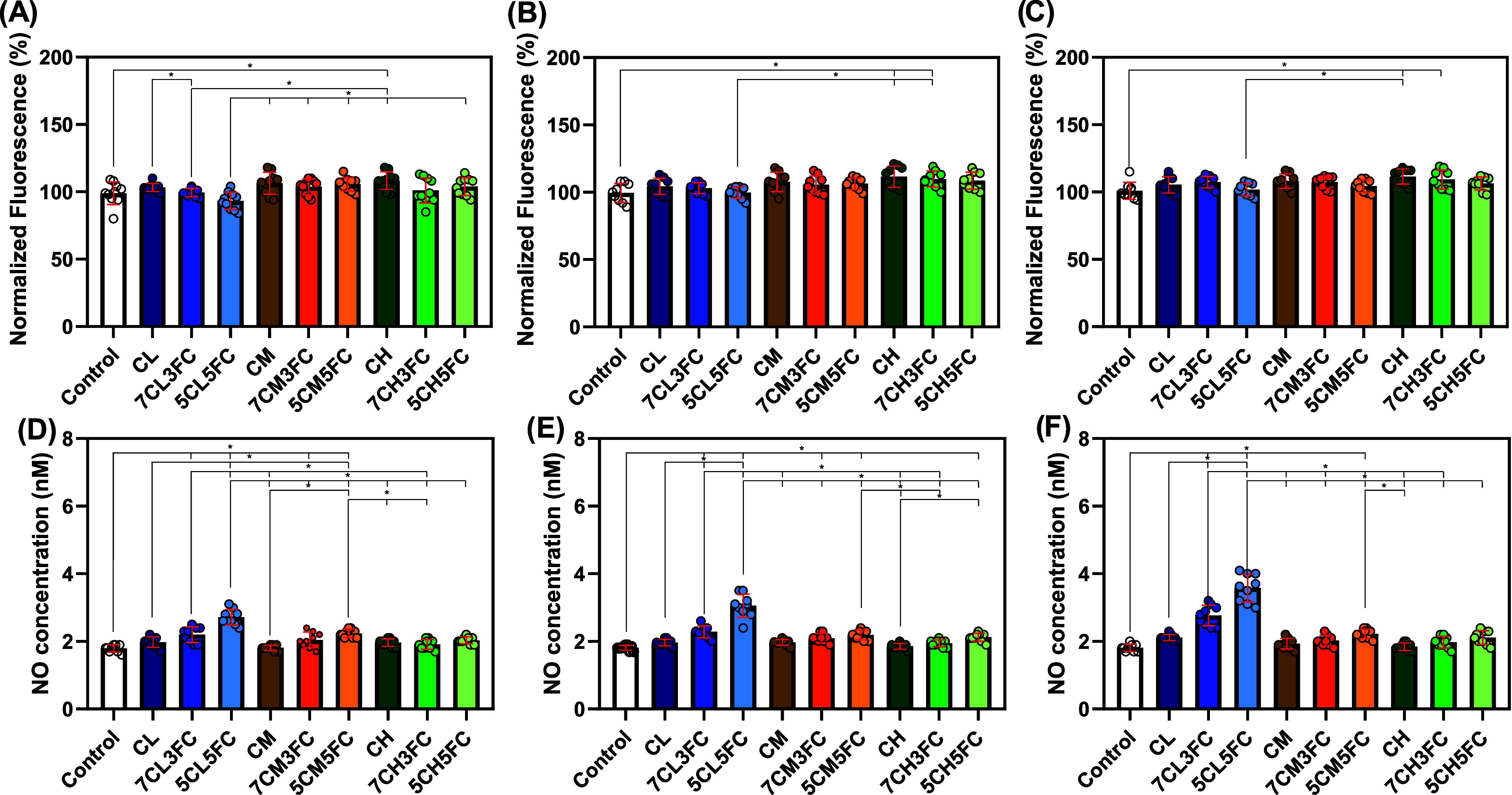
Intracellular oxidative and nitrosative stress in chitosan- and collagen-based samples. (A–C) Intracellular ROS levels assessed by the DCFDA assay at days 1, 3, and 7, respectively (values normalized to the control). (D–F) Intracellular RNS levels at days 1, 3, and 7, respectively. Results are presented as mean ± standard deviation (*n* = 9). Statistical significance was determined by one-way ANOVA with post hoc test, where *p < 0.05.

#### 3.2.4 Cell Migration

Cell migration was evaluated indirectly using the scratch wound assay performed with the conditioned medium obtained from the films (Figure 4). This approach allows assessing whether soluble components released from the materials, such as peptides and degradation byproducts, can influence fibroblast motility without the interference of surface topography.^59^ After 24 and 48 h, the untreated control group showed approximately 27% and 40% wound closure, respectively, establishing the baseline migratory capacity of the cells under standard culture conditions. The low-molecular-weight chitosan (CL) group displayed behavior similar to that of the control, indicating that chitosan alone does not significantly modulate fibroblast migration. In contrast, the addition of fish collagen produced a clear stimulatory effect: films containing 30% and 50% collagen (7CL3FC and 5CL5FC) reached ∼50% wound closure after 24 h and approximately 77% and 91%, respectively, after 48 h. For the medium-molecular-weight chitosan samples (CM), migration remained close to control levels at both time points; however, the presence of 30% or 50% collagen still improved closure rates modestly, achieving roughly 50% after 24 h and 69% after 48 h. This moderate response suggests that higher molecular weight and greater matrix density may reduce the release of diffusible bioactive components into the medium. These results are supported by the work of You et al. that investigates the migration capacity of fibroblast and keratinocytes cells using collagen/chitosan scaffolds loaded with silver nanoparticles.^60^ In contrast, the high-molecular-weight chitosan samples (CH) showed no significant differences compared with the control, even after collagen incorporation, further supporting the idea that dense, less permeable matrices hinder the diffusion of collagen fragments and growth-promoting molecules. These results demonstrate that collagen-derived factors present in the conditioned medium can enhance fibroblast mobility, likely through the activation of integrin-dependent signaling and the upregulation of matrix metalloproteinases that facilitate cell movement across the wound gap.

**Figure 4:**
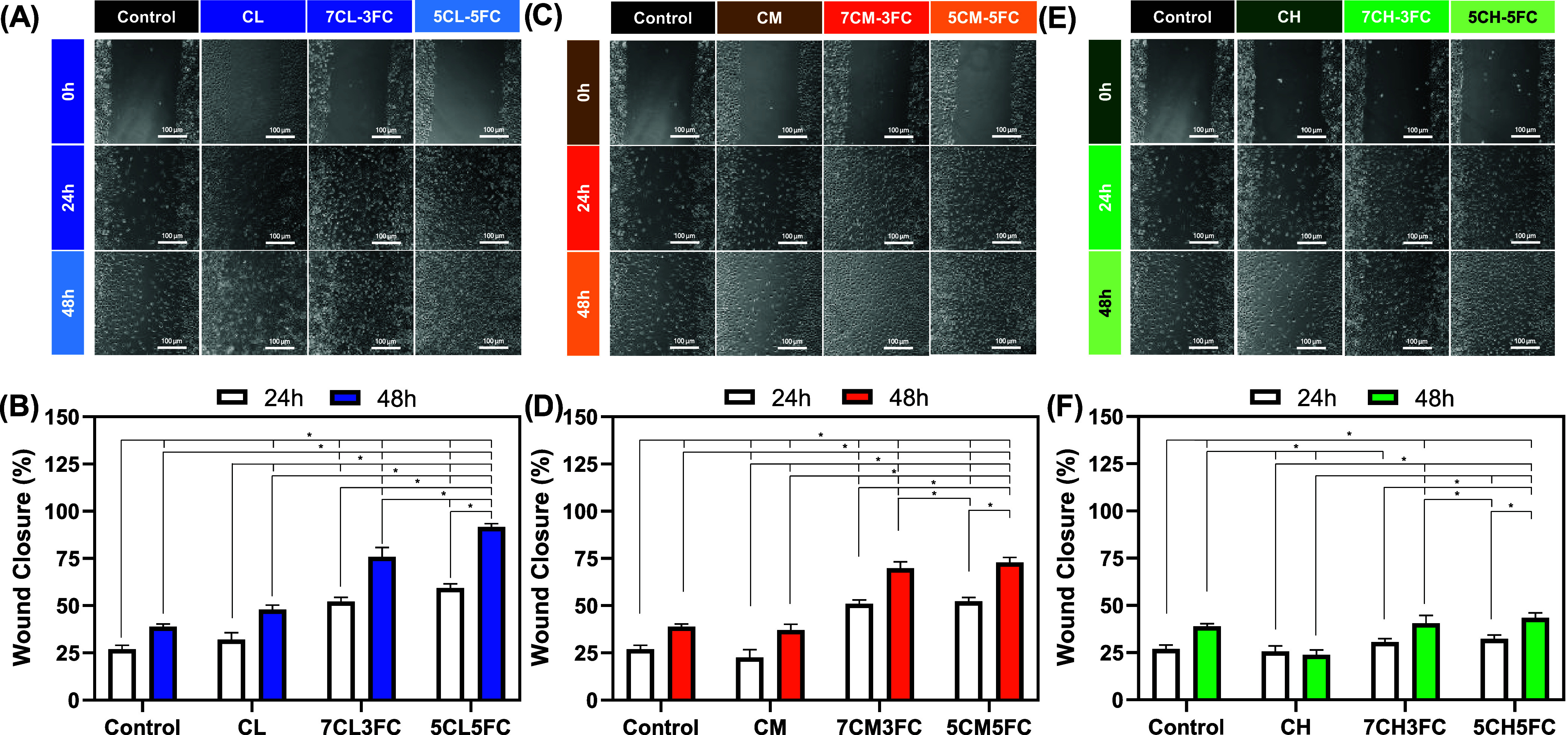
Cell migration assay of chitosan- and collagen-based samples. (A, C, E) Representative images of the wound-healing assay at different time points (24 and 48 h). (B, D, F) Quantification of wound closure corresponding to the images. Results are presented as mean ± standard deviation (*n* = 3). Statistical significance was determined by two-way ANOVA with post hoc test, where *p < 0.05.

#### 3.2.5 Cell Adhesion

Cell adhesion on the chitosan–fish collagen films was further investigated using confocal fluorescence microscopy after 3 days of culture with L929 fibroblasts (Figure 5). Phalloidin conjugated to Alexa Fluor 488 and DAPI were employed to visualize the cytoskeleton and nuclei, respectively, allowing detailed assessment of cell morphology and surface interaction. Across all samples, the confocal images revealed cells exhibiting elongated or partially stretched morphologies, characteristic of well-adhered fibroblasts initiating active spreading on the substrate. This indicates that none of the compositions interfered negatively with the anchoring process and that the materials provided a favorable microenvironment for cell attachment. These results are similar to those obtained by Li et al. and Liu et al. using chitosan/collagen scaffolds.^61,62^ Quantitative analysis of adhered nuclei per milligram of mm^2^ confirmed these observations. Incorporation of 50 wt % fish collagen led to a marked increase in cell adhesion, particularly for films based on low- and medium-molecular-weight chitosan. This enhancement can be attributed to the presence of collagen-specific peptide motifs, such as Gly–Pro–Hyp sequences, that promote integrin-mediated recognition and improve cell–matrix interactions.^63^ In contrast, the high-molecular-weight chitosan films, even when supplemented with collagen, exhibited cell densities comparable to those of the pure chitosan control. The denser polymeric network and reduced porosity likely limit surface exposure of collagen moieties and hinder the adsorption of serum proteins that mediate the initial adhesion.

**Figure 5:**
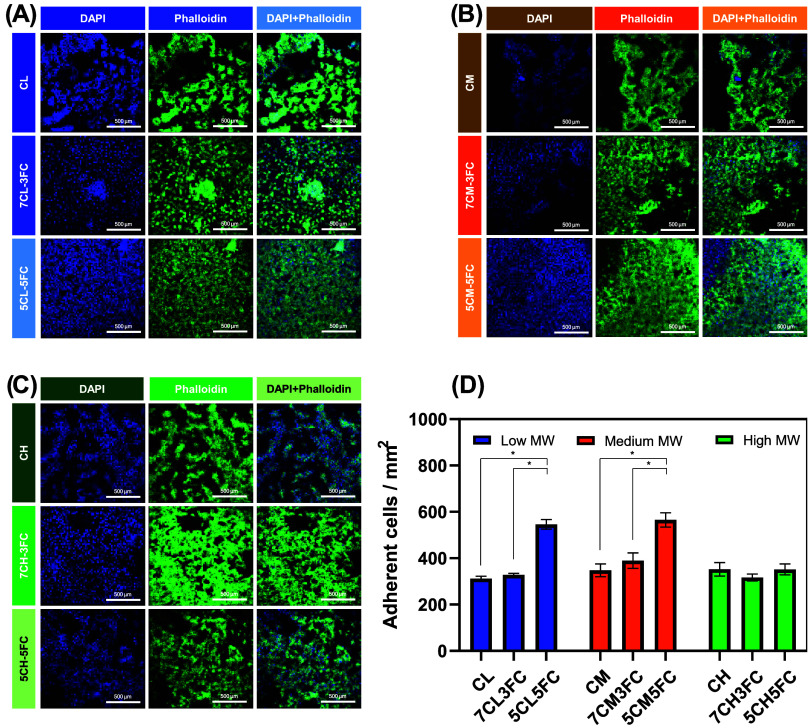
Cell adhesion on chitosan- and collagen-based films. (A–C) Confocal optical microscopy images of adhered cells after 3 days of contact, showing nuclear staining with DAPI and cytoskeleton organization with Phalloidin. (D–F) Quantification of adhered cells per mm^2^ after 1 day. Results are presented as mean ± standard deviation (*n* = 3). Statistical significance was determined by one-way ANOVA with post hoc test, where *p < 0.05.

#### 3.2.6 Hemocompatibility

The hemocompatibility of the chitosan–fish collagen films was evaluated through blood coagulation, assessment of red blood cell aggregation, and hemolysis rate to assess their suitability for contact with biological fluids (Figure 6). The blood coagulation time results are listed in Figure 6A. The control blood sample presented a coagulation time of approximately 7 min, whereas all film samples markedly shortened this period, demonstrating a clear pro-coagulant effect. Compared to the control, all film samples significantly reduced the coagulation time, confirming the intrinsic pro-hemostatic behavior of chitosan. This effect is commonly attributed to the cationic nature of chitosan amino groups, which promote electrostatic interactions with negatively charged erythrocyte membranes and platelet surfaces, leading to platelet adhesion, aggregation, and subsequent activation of the coagulation cascade.^64^ The incorporation of fish collagen produced distinct effects depending on the molecular weight of the chitosan matrix. In the low-molecular-weight chitosan films, the incorporation of 30% and 50% fish collagen led to a clear reduction in coagulation time, decreasing from around 6 min for pure chitosan (CL) to approximately 4 min and 40 s, representing a reduction of about 30–40% relative to the untreated blood sample. For the medium-molecular-weight formulations, the addition of collagen produced no significant variation with coagulation times remaining close to pure chitosan (CM). In contrast, the high-molecular-weight chitosan samples (CH) exhibited the opposite effect: when 50% collagen was incorporated, the clotting time increased from about 4 min and 30 s to nearly 6 min and 40 s, approaching the natural coagulation time of blood. These distinct trends can be explained by the structural and surface differences among the formulations. In the low-molecular-weight systems, collagen addition likely increases hydrophilicity and provides additional binding sites for plasma proteins, promoting faster fibrin network formation.^65^ The medium-molecular-weight chitosan already offers favorable surface charge and porosity for platelet activation, which explains the absence of major changes. Meanwhile, in the high-molecular-weight blends, the denser and less permeable structure may restrict plasma diffusion and reduce exposure of chitosan cationic sites, thereby limiting contact activation of coagulation factors.

**Figure 6:**
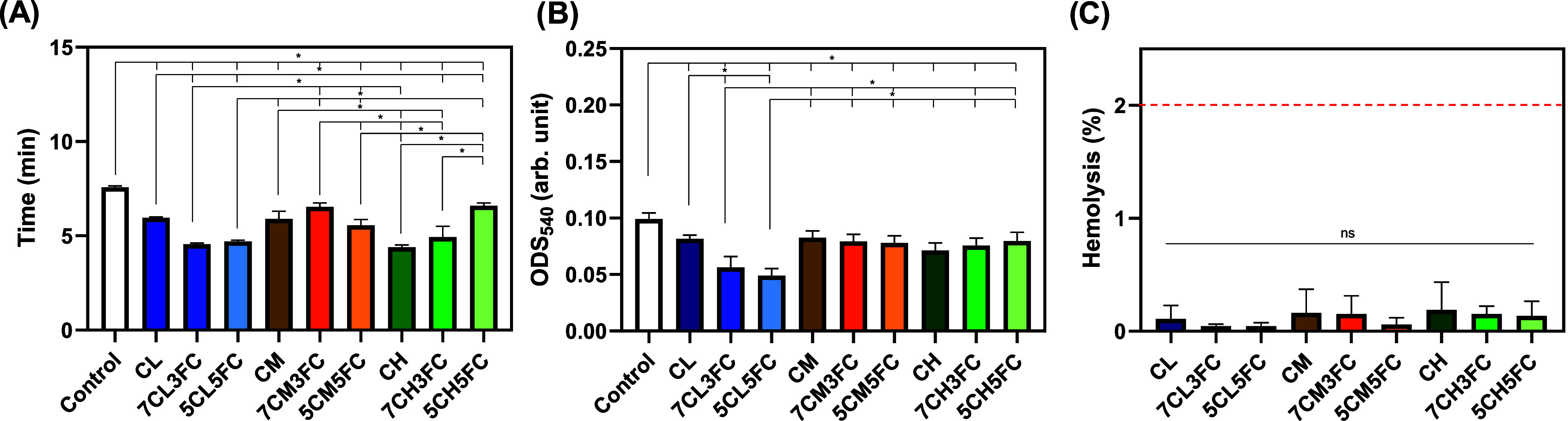
Hemocompatibility evaluation of chitosan- and collagen-based films. (A) Blood clotting time in contact with the films. (B) Quantification of free hemoglobin by absorbance measurement at 540 nm. (C) Hemolysis rate measured at 540 nm. Results are presented as mean ± standard deviation (*n* = 5). Statistical significance was determined by one-way ANOVA with post hoc test, where *p < 0.05.

In addition to the coagulation time, the amount of noncoagulated liquid was collected after clot formation, and the free hemoglobin content was quantified by optical density at 540 nm (Figure 6B). In general, all film samples significantly reduced the absorbance compared with the control without dressing (from 0.10 to 0.08), confirming lower levels of free hemoglobin and effective clot stabilization. Notably, the films containing 30% and 50% fish collagen combined with low-molecular-weight chitosan exhibited a further reduction, reaching absorbance values around ∼0.05. This result indicates a more efficient entrapment of erythrocytes and stabilization of the fibrin network, consistent with the faster coagulation behavior observed for these compositions. The hemolysis rate analysis confirmed excellent blood compatibility for all film formulations, with average values below 0.01% and no statistically significant differences among the samples (Figure 6C). This negligible hemolytic activity indicates that both chitosan and fish collagen are inherently nonhemolytic and maintain the structural integrity of erythrocyte membranes. According to ASTM F756-17, materials exhibiting hemolysis below 2% are classified as nonhemolytic, confirming the outstanding safety margin of the developed films. The mild surface charge of chitosan, together with the biocompatible and protein-like nature of collagen, likely contributes to minimizing electrostatic stress and preventing membrane rupture.^66,67^ These features are particularly advantageous for biomaterials designed for wound healing or direct blood contact as they ensure hemocompatibility, prevent inflammatory reactions, and preserve the physiological balance at the tissue–material interface.

#### 3.2.7 Genotoxicity

The genotoxic potential of the developed films was evaluated using the micronucleus assay, a well-established test for detecting chromosomal damage and mitotic spindle disturbances in eukaryotic cells.^29^ Assessing genotoxicity is essential in biomaterial studies as it determines whether prolonged contact with degradation products or extractables could induce DNA damage or genomic instability in surrounding tissues. In this study, none of the tested film extracts caused a statistically significant increase in the frequency of micronuclei when compared to the negative control, indicating the absence of clastogenic or aneugenic effects (Figure 7). In contrast, the positive control treated with mitomycin C exhibited a markedly higher incidence of micronuclei, confirming the validity and sensitivity of the assay. These findings are consistent with previous studies that reported the absence of genotoxic effects in collagen-based biomaterials. dos Santos Jorge Sousa et al. observed similar results when evaluating collagen extracted from *Paralichthys* sp.,^68^ while Wimalagunarathna and Gunathilake found no genotoxicity for collagen derived from *Decapterus macarellus*.^69^ Likewise, Seo et al. confirmed the genetic safety of collagen from *Raja kenojei*.^70^ Collectively, these results reinforce that the chitosan–fish collagen composite films developed in this work are nongenotoxic, biocompatible, and suitable for safe use in biomedical and regenerative applications.

**Figure 7:**
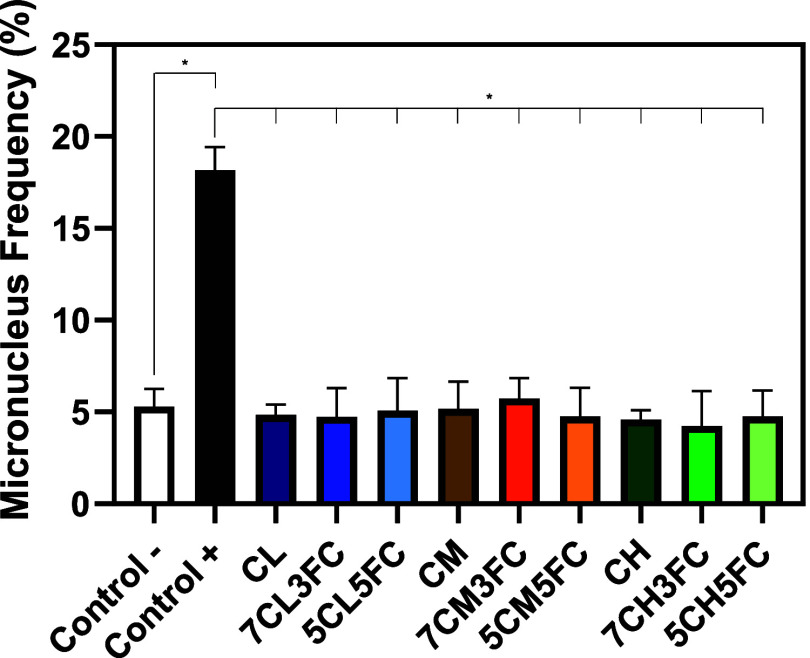
Micronucleus assay of the CHO-K1 cell line during the experimental period of 24 h. The results are presented as mean ± standard deviation (*n* = 3). Statistical significance was determined by one-way ANOVA with post hoc test, where *p < 0.05.

## 4 Conclusions

This work successfully demonstrates the development of bioactive, marine-derived composite films that combine ecological sustainability with high biological performance. Type I collagen from *M. furnieri* skin, a fish residue often discarded as waste, was effectively extracted and structurally preserved, proving to be a renewable and underexploited source of biomedical-grade collagen. Its incorporation into chitosan matrices of different molecular weights profoundly influenced the physical, structural, and biological responses of the composites. The disruption of chitosan’s crystalline domains, observed by XRD and DSC, resulted in softer and more amorphous films while maintaining sufficient cohesion for handling and application.

From a biological standpoint, the materials exhibited excellent cytocompatibility, supporting fibroblast adhesion, proliferation, and migration, all essential processes for wound healing. The modulation of intracellular redox balance, characterized by stable ROS levels and enhanced RNS generation in collagen-rich matrices, suggests that these composites may create a favorable microenvironment that promotes angiogenesis and tissue remodeling. Additionally, the marked reduction in the blood coagulation time without inducing hemolysis confirms their hemostatic potential and compatibility with direct blood contact. Among the tested formulations, the low-molecular-weight chitosan containing 50% collagen (5CL5FC) achieved the best overall performance, combining structural flexibility, cellular stimulation, and hemocompatibility. The films showed no genotoxic effects, confirming their safety for biomedical use. These results validate the synergistic role of collagen and chitosan in mimicking the extracellular matrix and demonstrate how marine biopolymers can be engineered into advanced materials that are both sustainable and clinically meaningful. The approach bridges marine resource valorization with regenerative medicine, paving the way for scalable, ecoefficient wound dressing technologies.

## Supplementary Material



## Data Availability

Data will be made available on request.

## Notes

Characterization of collagen by FTIR, confocal microscopy, and polarized light microscopy with picrosirius staining, and water uptake analysis (PDF)

## Abbreviations

XRD: X-ray diffraction
DSC: differential scanning calorimetry
FTIR: Fourier-transform infrared spectroscopy
ROS: reactive oxygen species
RNS: reactive nitrogen species
NaOH: sodium hydroxide
C_4_H_9_OH: butanol
H_2_O_2_: hydrogen peroxide
CH_3_COOH: acetic acid
NaCl: sodium chloride
DMEM: Dulbecco’s Modified Eagle’s Medium
FBS: fetal bovine serum
PFA: paraformaldehyde
DAPI: 4′,6-diamidino-2-phenylindole
PPP: platelet-poor plasma
PRP: platelet-rich plasma
TS: tensile strength
EB: elongation at break
NO: nitric oxide
sGC: guanylate cyclase
