# Supplementary material for: Green Marine Collagen–Chitosan Composites with Biocompatible, Hemostatic, and Pro-Healing Performance
Source: ACS Appl Bio Mater. 2026 Feb 26;9(6):3015–28. doi: 10.1021/acsabm.5c02493 (PMC12997167; doi:10.1021/acsabm.5c02493)
Supplement: Supplementary file 1 [file mt5c02493_si_001.pdf]

SUPPORTING INFORMATION

Green Marine Collagen-Chitosan Composites with Biocompatible, Hemostatic, and Pro-Healing Performance

Marcelo Assis[1,*], Diana Gabriela Nina Nina[1], Karolyne dos Santos Jorge Sousa[1], Mirian Bonifacio[1], Amanda de Souza[1], Mariana Carvalho Simões[1], Renata Granito[1], Flavia de Oliveira[1], Ana Claudia Muniz Rennó[1]

[1] Department of Biosciences, Federal University of São Paulo (UNIFESP), Santos-SP, 11015-020, Brazil.

* Corresponding author: marcelostassis@gmail.com

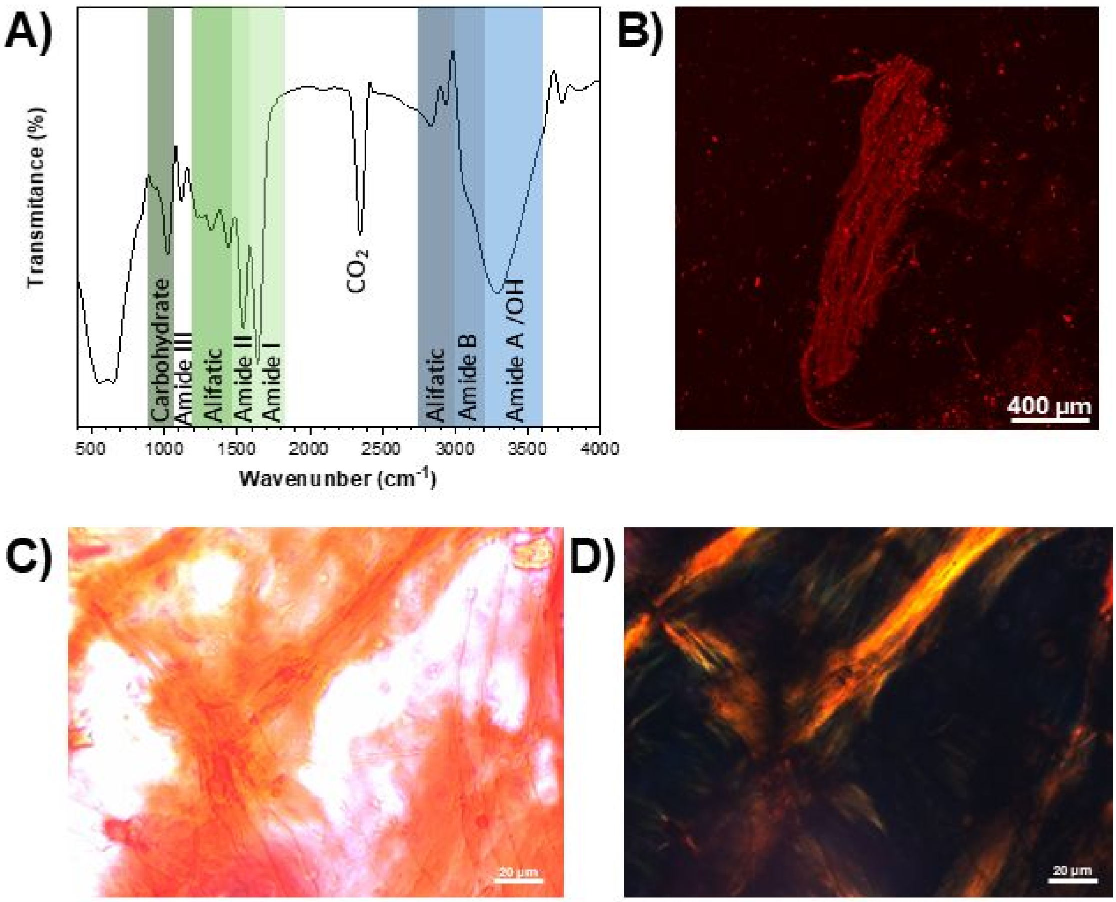

Figure S1. Fish collagen characterizations. (A) FTIR spectrum. (B) Confocal microscopy using rhodamine B as fluorophore. (C) Light microscopy and (D) polarized light microscopy of fish collagen sample using picrosirius stained.

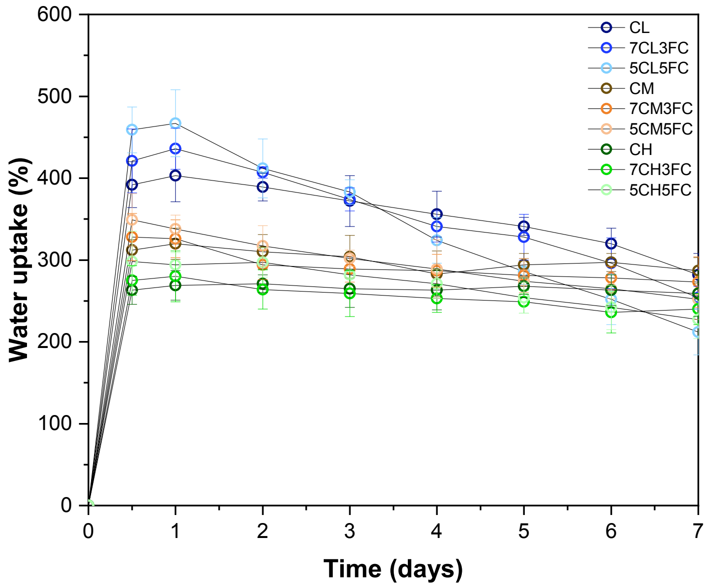

Figure S2. Water uptake of the composite samples.
